# The association between labour variables and primiparous women’s experience of childbirth; a prospective cohort study

**DOI:** 10.1186/1471-2393-14-208

**Published:** 2014-06-18

**Authors:** Hanna Ulfsdottir, Eva Nissen, Elsa-Lena Ryding, Doris Lund-Egloff, Eva Wiberg-Itzel

**Affiliations:** 1 Department of clinical science and education, Section of Obstetrics and Gynaecology, Karolinska Institute, Soder Hospital, Stockholm, Sweden; 2 Department of Women’s and Children’s Health, Karolinska Institute, Stockholm, Sweden; 3 Department of LabMed, section of Clinical Chemistry, Karolinska Institute, Huddinge Hospital, Stockholm, Sweden

**Keywords:** Adverse foetal outcome, Amniotic fluid lactate, Dystocia, Experience of childbirth

## Abstract

**Background:**

Studies have suggested several risk factors for a negative birth experience among primiparas. Factors that are mentioned frequently include labour dystocia, operative intervention such as acute caesarean section or vacuum extraction, or the infant being transferred to neonatal care. Another important factor mentioned is lack of support from the midwife.

**Methods:**

A study was made of the deliveries of 446 healthy primiparas in a prospective cohort study performed at Soder Hospital, Stockholm, Sweden. Samples of amniotic fluid were collected at delivery and the levels of amniotic fluid lactate (AFL) were measured to give an indication of the metabolism of the uterine tissue. Obstetrical data were collected from birth records.

Postpartum, all the women included in the study were asked to complete the Wijma Delivery Experience Questionnaire (W-DEQ B) that measures the experience of a woman’s delivery. The main objective of the project was to study well-known as well as new factors associated with negative experience of childbirth among a group of healthy primiparas.

**Results:**

Risk factors for reporting a higher level of negative childbirth experience were shown to be a high level of AFL (AOR 3.1, 95%, CI; 1.1-8.9), a longer latent phase (AOR 1.8, 95%, CI; 1.03-3.1), and a low Apgar score (<7 at 1 min) (AOR 13.3, 95%, CI; 1.6-111.0). Those women who had a negative birth experience wanted the midwife to be present more of the time during labour (*p = 0.003*).

**Conclusions:**

A high AFL level, as a marker of uterine metabolic status, and a longer latent phase are strongly associated with a negative experience of childbirth. A low 1 minute Apgar score of the newborn seems to have the strongest negative influence on the woman’s experience of childbirth, even when the infant recovers immediately.

## Background

Giving birth is a complex event. A woman’s experience of childbirth is influenced by her psychological and social situation as well as by cultural conditions. A good experience of labour is important for the woman’s wellbeing, for her relationship with her baby and for breastfeeding, as well as for her planning of future pregnancies [1-3]. A negative birth experience [4-6] may lead to a desire for a planned caesarean section in subsequent pregnancies. Previous studies have suggested several risk factors for a negative birth experience. The factors often mentioned include labour dystocia, operative intervention such as acute caesarean section or vacuum extraction, lack of support from the midwife, and the infant being transferred to neonatal care [7-16]. High levels of maternal stress and anxiety have been shown to influence the progress of labour with higher levels of epinephrine being associated with a lower uterine contractile activity as measured by Montevideo units [17].

When activity intensity is high, the body has to produce energy quickly. Energy can be produced through aerobic as well as anaerobic metabolism. The production of lactate is the final step in the glycolytic pathway where pyruvate is broken down to form energy. The concentration of lactate can increase more than tenfold in physical exertion but the rise is of short duration and depends on the oxygen supply to the tissue. One consequence of this is that a particular tissue can produce lactic acid during exertion while several other tissues may still have a good supply of oxygen.

Previous studies have shown that the level of lactate in the uterine tissue increases during labour contractions as a sign of anaerobic metabolism. In abnormal contractions, the removal of lactate appears to deteriorate and lactate and other metabolites will be accumulated. In these conditions, the power of the uterine contractions decreases and labour progress is halted. The level of lactate in the uterine muscle is mirrored in the level of lactate in amniotic fluid (AFL), and can easily be analysed at the bedside [18-20]. No previous investigation has been made of whether the AFL level has any association with the woman’s experience of childbirth.

An a priori hypothesis of the study was that labour dystocia, defined as cervical dilation slower than 1 cm per hour or a delayed progress beyond two hours, is associated with a higher level of negative childbirth experience among recently delivered, primiparous women. The study also aimed to explore the association of the outcome variable with other possible risk factors, including an elevated AFL level.

## Methods

A prospective observational study called `the dysfunctional labour study´ was performed at Soder Hospital Stockholm, Sweden between April 2010 and January 2012.Healthy, Swedish-speaking, nulliparous women, with a singleton pregnancy and a gestational age between 37–42 weeks, were asked to participate. A number of criteria for inclusion were formulated: labour should have started spontaneously, the foetus should be in a cephalic presentation, the cervical dilation should be at least 3 cm and regular, painful contractions should be present.

A group of 25/140 midwives at the Soder Hospital clinic volunteered to assist in data collection and informed the labouring women about the ongoing study. In all, 561 healthy primiparous women in labour, presenting consecutively, were invited to participate in this study as they fulfilled the inclusion criteria. Of these, seven declined to participate and 107 were excluded later due to incomplete ID or an incomplete questionnaire. One woman was excluded due to intrapartum death of the infant. The remaining 446 women constituted our material for the survey. A written informed consent for participation in the study was obtained from all participants. The study was approved by the Karolinska Institute Human Research Ethics Committee (2010/199-31/1).

All women included were asked to complete the *Wijma Delivery Experience Questionnaire* (W-DEQ B) the day after delivery. The W-DEQ B measures the woman´s experience of childbirth after delivery. The questionnaire includes 33 items, each scoring from 0–5. The sum score ranges from 0–165; the higher the score, the worse the woman’s experience. The questionnaire has shown good psychometric properties [21]. The cut-off between good/moderate and negative experience of childbirth was set at a sum score of 66, which was the 75^th^ percentile in the original study of childbirth experience [21]. The two groups (W-DEQ < and ≥ 66) were compared with regard to levels of AFL, and labour variables in several parameters.

In addition to the W-DEQ, seven questions were added relating to nourishment, sleep, the presence of the midwife during labour and thoughts about a subsequent pregnancy and delivery. In calculating the outcome, the same scale was used as in the original W-DEQ B questionnaire, with a scoring range from 0–5.The questionnaire was collected after delivery but before the woman was discharged from hospital.

In order to detect labour dystocia, the WHO-modified partogram was used with an alert line and a two hours displaced action line, as recommended in Sweden at the time of the study. The alert line was passed if the cervical dilation was slower than 1 cm per hour, and the action line was passed if the delay in progress continued beyond two hours.

In line with the study guidelines, a sample of amniotic fluid was collected by the midwife at delivery. The level of lactate in AF was used as a sign of the uterine metabolic status at delivery and the analysis was performed immediately by a research midwife, using a lactate measurement device, (LMU061, ObsteCare AB, Sweden) [18-20]. The test result was blinded to both the midwife and the labouring woman. A cut-off value for a very high AFL level was set at ≥ 12 mmol/L. This is in line with earlier publications where there was an increased frequency of complicated deliveries in the group with a high AFL value [18-20].

Foetal scalp blood sampling was performed in the deliveries where a non-reassuring/pathological CTG (Cardiotoccography) trace was presented. The test was performed with the mother supine and her legs in stirrups. The scalp blood was collected in preheparinized glass capillary tubes, and the lactate analysis was carried out at the bedside.

After delivery and before the newborn’s first cry, arterial and venous cord blood samples were drawn from a segment of the cord and analysed within a few minutes for ph. All cord blood analyses were performed using an ABL 800 analyser (Bayer®) available in the labour ward. Socio-demographic and obstetric background data were collected from the antenatal and delivery records.

Statistical analyses were performed using SPSS 20.0 (SPSS Inc. Chicago, Illinois, USA) and the Statistica for Windows statistical package, version 10.0 (Stat Soft, Tulsa, Oklahoma, USA). Mean (m) and standard deviation (SD) were used as descriptive measures. The difference between the groups of low/moderate or high W-DEQ (<or ≥ 66p) was tested by t-test for parametric variables and chi-square test for categorical variables (%). The sum of the responses on the W-DEQ questionnaire was calculated. Internal missing data were replaced by the group mean for the unanswered question, according to the guidelines for the survey. The scores from the questionnaire were then compared with the outcome of delivery. A logistic regression [22] was performed to estimate the association between the primary outcome (W-DEQ < or ≥ 66p) and the following independent variables: labour dystocia (yes/no), latent phase >13 h (yes/no), full cervical dilation and non-descent of the foetal head >3 h (yes/no), AFL ≥ 12 mmol/L (yes/no), epidural anaesthesia (yes/no), Apgar < 7 at 1 min (yes/no). Our model strategy was as follows: first, unadjusted associations with each factor were studied in a univariate model. Second, the adjusted association with respect to the risk factors measured was studied in a multivariable model. Before performing the logistic regression, clinically relevant interaction models were constructed (such as high lactate and low Apgar score). No significant interactions were detected.

## Results

In all, 446 healthy women were included in this study of various factors that might affect women’s experience of delivery. After all the women had completed the W-DEQ B questionnaire, four of the questions remained unanswered. According to the guidelines for the questionnaire, internal missing data could be replaced by the group mean for the unanswered question, which has been made.

44% (152/446) of the women belonged to the group with a W-DEQ score ≥ 66, which corresponds with a higher degree of negative experience during delivery. The background characteristics of the women included are presented in Table 1. The mean age of the women was 30 years. No differences were presented according to BMI (Body Mass Index), height and education. Smoking before pregnancy was more common in the group with a higher W-DEQ score.

**Table 1:** Background characteristics for the 446 women included

	**W-DEQ <66 (n = 294)**	**W-DEQ ≥ 66 (n = 152)**	P- value*
Maternal age (years)	30.2 (4.3)	30.1 (4.7)	0.95
BMI^^^	23.6 (8)	23.6 (8)	0.97
Height (cm)	167.2 (8.9)	166.8 (9.8)	0.64
Smoker before pregnancy (n)	36 (12.2)	27 (17.8)	0.037*
Education- primary school	26 (8.8)	16 (10.5)	0.4
High school	103 (35)	60 (39.5)	0.4
College/University	165 (56.1)	75 (50)	0.4

^^^BMI = weight/(length* length).

*P-values <0.05 were considered as statistically significant.

Data are presented as numbers (%) or mean (SD).

The difference between the groups was tested by t-test for parametric variables (SD) and chi-squared test for categorical variables (%).

Labour outcomes are presented in Table 2. No differences were shown in the method of delivery. Dystocia according to the partogram was not associated with a negative experience of childbirth. Nor were any differences found in the length of the first or second stage of labour. However, a significantly longer latent phase was shown in the group with a W-DEQ score ≥ 66p (8.02 h vs. 9.59 h). A high W-DEQ score more often followed labours with an AFL level ≥ 12.0 mmol/L (12 vs. 6%) and with the use of epidural anaesthesia (75% vs. 61%). The use of oxytocin to augment labour did not differ significantly between the two groups.

**Table 2:** Labour outcome of the 446 women included

	**W-DEQ <66 (n = 294)**	**W-DEQ ≥ 66 (n = 152)**	P- value*
Dystocia (n)	129.0 (43.9)	75.0 (49.3)	0.32
Latency phase (h)	8.02 (5.54)	9.59 (6.34)	0.002*
First stage (h)	11.28 (4.50)	11.23 (5.14)	0.87
Mode of delivery			
Spontaneous vaginal (n)	233.0 (79.3)	107.0 (70.4)	0.14
Ventouse (n)	36.0 (12.2)	27.0 (17.8)	0.14
Caesarean (n)	25.0 ( 8.5)	18.0 (11.8)	0.14
Pushing (min)	37.0 (20.8)	37.6 (23.0)	0.78
AFL** ≥12 mmol/L (n)	6.0 (2.7)	12.0 (9.8)	0.009*
EDA (n)	179.0 (60.8)	114.0 (75)	0.002*
Use of Oxytocin ≥60 min (n)	137.0 (80.6)	93.0 (89.4)	0.06
Haemorrhage ≥1000 ml (n)	22.0 (8.3)	6.0 (4.5)	0.21
Sphincter rupture Degree 3 or 4 (n)	16.0 (5.4)	14.0 (9.2)	0.16
Fetal scalp blood sampled (n)	44.0 (15)	25.0 (16.4)	0.68

*P-values <0.05 were considered as statistically significant.

**AmnioticFluidLactate.

Data are presented as numbers (%) or mean (SD).

The outcome of the infant is shown in Table 3. No differences were found in gestational age, birth weight, height or head circumference of the baby. Girls were more common in the group with a lower W-DEQ score. A low Apgar score at 1 and 5 minutes after delivery, and a transfer of the newborn to the neonatal care unit were significantly more common in the group where a high W-DEQ score was reported.

**Table 3:** Outcome at delivery of the 446 newborns delivered in the study

	**W-DEQ <66 (n = 294)**	**W-DEQ ≥ 66 (n = 152)**	P- value*
Gestational days (n)	281.4 (7.5)	282 (7.3)	0.53
Weight (g)	3570 (413)	3596 (416)	0.53
Length (cm)	51.1 (1.8)	51.1 (1.9)	0.79
Head circumference (cm)	34.9 (1.3)	35 (1.4)	0.28
Girl (n)	159 (54.1)	66 (43.4)	0.052
Apgar score <7			
1 min	5 (1.7)	11 (7.2)	0.005*
5 min	0 (0.0)	3 (2.0)	0.04*
10 min	0 (0.0)	1 (0.7)	0.34
Transferred to neonatal intensive care unit (n)	8 (2.7)	12 (7.9)	0.026*
Arterial pH	7.25 (0.09)	7.25 (0.1)	0.67

*P-values <0.05 were considered as statistically significant.

Data are presented as numbers (%) or mean (SD).

The additional questions in the questionnaire (Table 4) showed a significant difference between the two groups in that women in the high W-DEQ group were less satisfied than those in the low group with the amount of time the midwife spent in the delivery room during labour. Many of the women in the high W-DEQ group also expressed a wish for the midwife to be present more of the time during labour. Fewer women in the high W-DEQ group would consider having another vaginal birth, or even having more children. Three additional questions about sleeping, eating and whether the woman had been looking forward to the delivery showed no significant differences between the groups regarding the W-DEQ score (results could be shown on request).

**Table 4:** Result of additional questions from the included women

	**W-DEQ B <66 (n = 294)**	**W-DEQ B ≥ 66 (n = 152)**	P- value*
Do you think that your midwife was present enough? (0 = no -5 = yes)	4.6 (0.8)	4.3 (1.0)	0.003*
Did you want your midwife to be present more (0 = no -5 = yes)	0.72 (1.3)	1.5 (0.12)	0.003*
Do you want to have more children?			
Yes	242 (82.3)	102 (67.1)	0.001*
No	5 (1.7)	5 (3.3)	
Don´t know	43 (14.6)	45 (29.6)	
Can you imagine a vaginal delivery next time?			
Yes	225 (76.5)	68 (44.7)	<0.001*
No	9 (3.1)	23 (15.3)	
Don´t know	57 (19.4)	59 (38.8)	

*P-values <0.05 were considered as statistically significant.

Data are presented as mean (SD).

In the multivariable logistic regression analysis (Table 5), the AFL - value and obstetric factors presented in previous publications as having an association with a negative delivery experience were analysed for their association with a high W-DEQ score. A high AFL level (≥12 mmol/L) at delivery, an extended latent phase, the use of epidural anaesthesia and an Apgar score < 7 at 1 min after delivery all seemed to be risk factors for negative experience of childbirth in this study. No significant association between a high W-DEQ score and labour dystocia according the partogram, nor mode of delivery, was indicated in this material. Nor was there any clear association with any of the other obstetrical variables studied.

**Table 5:** Associations between possible risk factors and W-DEQ B score ≥ 66

**Risk factors for W-DEQ ≥ 66**	**W-DEQ ≥ 66/total**	**OR unadjusted (95% CI)**	**AOR adjusted (95% CI)**
**Latency phase >13 h**			
No	100/333 (30%)	Ref	
Yes	52/112 (46%)	2.1(1.3-3.2)*	1.8 (1.03-3.1)*
**Labour dystocia**			
No	77/242 (32%)	Ref	
Yes	75/204 (37%)	1.2 (0.8-1.8)	
**Full cervical dilatation no descendence of foetal head >3 h**			
No	85/227 (37%)	Ref	
Yes	66/215 (31%)	1.3 (0.9-1.9)	
**Lactate In AF >12.0 mmol/L**			
No	110/325 (34%)	Ref	
Yes	12/18 (67%)	3.9 (1.4-10.7)*	3.1 (1.1-8.9)*
**Epidural**			
No	37/152 (24%)	Ref	
Yes	115/294 (39%)	2.0 (1.3-3.1)*	1.2 (0.7-2.0)
**Apgar score <7 at 1 min**			
No	141/428 (33%)	Ref	
Yes	11/16 (69%)	4.5 (1.5-13.1)*	13.3 (1.6-111)*

*P-values <0.05 were considered as statistically significant.

Values are expressed as Odds Ratio (OR) with corresponding 95% confidence intervals (CI).

According to the multivariable analysis, high AFL value (AOR 3.1, 95%, CI; 1.1-8.9) and a longer latent phase (AOR 1.8, 95%, CI; 1.03-3.1) seemed to be independent risk factors for having a high W-DEQ score among these 446 healthy primiparas. A low Apgar score at 1 minute after delivery also increased the risk of a negative childbirth experience (AOR 13.3, 95%, CI; 1.6-111.0).

## Discussion

In this prospective observational study of primiparas’ experience of childbirth, a high AFL level (≥12 mmol/L) and a long latent phase of labour seemed to be associated with a negative labour experience. A low Apgar score of the newborn at delivery was a further factor that strongly contributed to a high W-DEQ score.

Our main hypothesis was that labour dystocia would be associated with a negative experience of childbirth, but this was not confirmed by the study results. The results may contradict other studies describing a correlation between labour dystocia and a poor labour experience for the woman [8,10,11,23]. The definition of a dystocic labour in this study is set at two hours delay from the alert line in the partogram, as these were the guidelines in Sweden at the time of the data collection. As the definition of dystocia may be broad, T-tests were also performed with an extended labour progress as outcome (first stage > 17 h), [24] but no differences were identified with this definition either.

In general, most women are still at home during the latent phase, as they are advised not to come to the delivery ward `too early’. This causes the latent phase to be a time of fear, anxiety and fatigue. This may be one of the main reasons why a longer latent phase is to a high degree associated with a negative experience, shown by a high W-DEQ score in this study. A new and very interesting finding in this study is the association shown between a high W-DEQ score and a high AFL value at delivery. In earlier publications, around 10% of deliveries have been shown to have a high level of AFL already when attending the delivery ward. In many of these deliveries, a long latent phase is also presented [18-20]. With a long latent phase and a high AFL value, the woman probably experiences more painful and ineffective contractions for a very long time.

Lactic acid is mainly produced by anaerobic metabolism, when the uterine muscle receives insufficient oxygen. The AFL level is as a good marker of uterine metabolic status during labour and the production of lactate from the uterus may increase in situations of fear, anxiety and fatigue as a sign of poor oxygenation of the tissue [18-20]. This is something completely new, and should be followed up in subsequent studies. One of the questions to be answered in the future is whether the group of women with a negative labour experience also has a higher degree of abnormal uterine lactate production from the beginning, or whether the uterus produces an abnormal amount of lactate during ongoing labour.

Another interesting finding in this study was the increased use of epidural anaesthesia (EDA) in the group with a high postnatal W-DEQ score. Lactic acid is produced by anaerobic metabolism mediates ischemic pain and occurs when there is insufficient blood flow for the metabolic needs of an organ. The pain of a heart attack is the prototypical example. Multiple compounds released from the tissue probably contribute to the discomfort during a heart attack by acting on sensory neurons. The question is whether the uterine muscle reacts in the same way. Earlier publications have shown that the use of an epidural does not have a positive effect on the subsequent childbirth experience [8,23,25-29]. In a randomised controlled trial, no differences in the woman’s satisfaction with her experience of childbirth were found following the use of EDA [23]. Goodman et al. suggest that if women are able to manage their labour pain, they will be more satisfied with their own effort and therefore more positive about their total childbirth experience. This may also explain the higher numbers of women with a positive experience in the group without EDA and a low level of lactate in AF in this material.

Giving birth to a baby with a low Apgar score at 1 minute after delivery may be dramatic even though the baby may recover within a few minutes. The Apgar score at 1 minute after delivery was selected in this study for the logistic regression since the focus of the study is the mother´s postnatal experience of childbirth, not whether the baby is affected in the long term. It is obvious that giving birth to a baby with a low Apgar score is one of the most frightening experiences for a woman, even if the baby recovers rapidly.

Some limitations of this study should be mentioned. Soder Hospital is a large city hospital in Stockholm and the women included in the study were older and better educated than the national average in Sweden, which may have some influence on the findings. The numbers of women participating were also rather small, and constituted only a minor proportion of all 4,333 healthy primiparous women delivered at the clinic at gestational age 37–42 weeks during the time of the study. The reason is that the participation of the midwives in this project was voluntary and only a limited group of midwives with an interest in the project took part and included women in the study. The normal delivery rate was, however, the same (76%) in the study material as in the whole group of 4,333 women at the clinic during the period of the study.

The point in time when the women filled in the questionnaire can also be discussed. It is possible that women, only one day after delivery, are overwhelmed by having a healthy baby and the negative feelings from the delivery may only emerge later. However, such an interpretation is unlikely since an earlier prospective study of women with various types of deliveries showed that lower W-DEQ scores were found at one month than at two days postpartum [30]. The choice of a cut-off at W-DEQ B score 66 p may perhaps also affect our results. In order to study women with a really severe experience of childbirth, it will be necessary to use a higher cut-off point. The limited material of the present study, and our wish to consider not only a traumatic delivery but a moderately negative experience, dictated our choice of cut-off point. It is interesting that even with the low cut-off studied, more women with a high W-DEQ B score would not consider further pregnancies, or another vaginal delivery in the future; at least this was their position immediately after birth.

This study raises new and interesting questions about the influence of the metabolic status of the uterus on women’s experience of childbirth. Better management of the latent phase and/or more sensitive reassurance of women whose babies have a low Apgar at 1 minute could also make a difference. Of course, we do not know whether women with a high postnatal W-DEQ B score have other psychological vulnerabilities that may influence their decisions. In any event, it is clear that an extended latent phase in combination with an anaerobic metabolism of the uterus is strongly associated with a negative childbirth experience for a first-time mother. The findings of this study have to be replicated in future studies in other settings to determine whether this new knowledge would be helpful in future obstetrical clinical care.

## Conclusion

In this study we found that high AFL levels at delivery, as a sign of an aerobic metabolism in the uterine tissue, and an extended latent phase will associate with a negative experience of labour among healthy primiparas and with the women’s postnatal experience of childbirth.

### Availability of supporting data

Supporting data have been deposited at Karolinska Institute, Soder Hospital and can be shown on request.

## Competing interest

Each author represents and warrants that she has no financial affiliation (e.g., employment, direct payments, stock holdings, retainers, consultantships, patent-licensing arrangements, or honoraria) or involvement within the last 3 years with any commercial organisation with a potential financial interest in the subject or materials discussed in the manuscript.

## Authors’ contributions

EWI initiated the study and EWI, HU and DEL collected the data. EWI, HU, EN and ELR performed the data analyses. EWI, HU, EN, DEL and ELR drafted the manuscript and EWI, HU, EN, ELR, and DEL contributed all to the revision of the manuscript and approved the final version.

## Pre-publication history

The pre-publication history for this paper can be accessed here:

http://www.biomedcentral.com/1471-2393/14/208/prepub
